# Ultrasensitive ctDNA monitoring reveals early predictors of immunotherapy response in advanced cancer

**DOI:** 10.1038/s41698-026-01287-3

**Published:** 2026-01-24

**Authors:** Daisuke Nishizaki, Allison Law, Bailiang Li, Charles Abbott, Yi Chen, Suzanna Lee, Rachel Pyke, Kathleen Keough, Gregory A. Daniels, Kay T. Yeung, Sean M. Boyle, Richard O. Chen, Shumei Kato

**Affiliations:** 1 Center for Personalized Cancer Therapy and Division of Hematology and Oncology, Department of Medicine, University of California San Diego, Moores Cancer Center, La Jolla, CA USA; 2 Personalis Inc, Fremont, CA USA

**Keywords:** Biomarkers, Cancer, Computational biology and bioinformatics, Oncology

## Abstract

Circulating tumor DNA (ctDNA)-based response assessment is appealing but limited by conventional analytical thresholds. We utilized a whole genome sequencing based, tumor-informed ultrasensitive ctDNA assay which tracked ~1800 somatic mutations to analyze 227 longitudinal plasma samples from 39 patients with advanced/metastatic cancers receiving immune checkpoint inhibitors (ICIs). ctDNA was detected from 2.0-239,315 PPM (median limit of detection: 1.77 PPM), with 33% of positive detections below 100 PPM. Early molecular response, defined as >50% ctDNA reduction or sustained ctDNA negativity from baseline to first follow-up, strongly predicted improved progression-free survival (PFS) (hazard ratio (HR) = 0.09, 95% CI: 0.02-0.39, p = 0.001) and was independently prognostic of PFS. Molecular complete response (mCR), defined as any ctDNA clearance, predicted overall survival and PFS, with 1-year PFS of 87% in mCR patients versus 16% in non-mCR patients (HR = 0.14, 95% CI: 0.04-0.50, p = 0.003). The high-sensitivity ctDNA monitoring may enable precise, real-time evaluation of ICI response to guide clinical decision-making.

## Introduction

The rapid evolution of immuno-oncology (IO) therapies and combinations has created an urgent need for more precise methods to guide treatment decisions and monitor therapeutic response. Current predictive biomarkers, including microsatellite instability (MSI) status^1^, PD-L1 expression^2^ and tumor mutation burden (TMB)^3^, have shown limitations in predicting treatment response and duration of response, and conventional imaging techniques may not accurately assess therapeutic outcomes, particularly in cases of pseudoprogression^4,5^. This limitation is particularly evident in the metastatic disease setting, where rapid clinical decision-making and treatment optimization is required to improve patient outcomes^6^.

Accurate and early determination of immunotherapy response status is fundamental to optimizing patient outcomes through informed treatment decisions. Assessment of therapeutic effectiveness prior to conventional radiographic evaluation could enable critical, time-sensitive modifications to treatment regimens, including therapeutic switches or intensification protocols^7,8^. Moreover, early response indicators could prevent the clinical consequences of continued ineffective therapy, protecting patients from excessive exposure to potentially toxic agents. However, the paucity of methods that effectively monitor treatment response in a real-time fashion continues to impair treatment-making decisions.

Circulating tumor DNA (ctDNA) analysis has emerged as a promising solution for real-time, non-invasive monitoring of tumor dynamics during immune checkpoint blockade (ICB)^9^, enabling rapid identification of primary resistance^10^ and timely therapeutic adjustments^11^. This molecular response-driven approach, utilizing minimally invasive dynamic ctDNA monitoring, presents an opportunity to optimize therapeutic outcomes while minimizing the likelihood of patient adverse events. Despite this promise, clinical uptake of ctDNA assays has been constrained by the limited sensitivity of early approaches, which were largely conducted using techniques with detection limits above 80-100 parts per million (PPM)^12–14^. This barrier to implementation is reflective of unanswered clinical questions: optimal methods for quantifying ctDNA levels; the relationship between ctDNA features and survival outcomes; the definition and timing of molecular response; correlation with Response Evaluation Criteria in Solid Tumors (RECIST) criteria; and identification of patient populations most likely to benefit from molecular response assessment.

In the present study, we sought to address these limitations by evaluating an ultrasensitive personalized ctDNA assay capable of detecting ctDNA down to 1-3 PPM, unlike prior studies, which were constrained by higher detection thresholds. We applied this approach across a pan-cancer cohort of patients with treatment-refractory, metastatic/advanced disease undergoing IO treatment. This study investigated ctDNA kinetics at early on-treatment timepoint (median=23 days after IO initiation), enabling assessment of molecular response well before conventional radiographic evaluation.

## Results

### Patients and ctDNA analysis

**Fig. 1:**
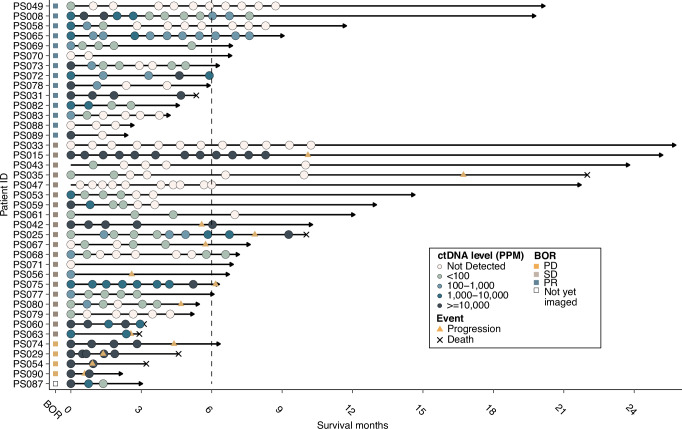
Overview of longitudinal patient timeline and plasma sampling (*N* = 39). Swimmer plot of longitudinal ctDNA status and clinically significant events, i.e. disease progression, and either mortality or most recent follow-up evaluation. Patients are arranged by best overall response (RECIST). Boxes at the origin of each swimmer plot display data for corresponding best overall response for each patient. Line length for each patient track indicates duration of overall survival from treatment start.

Plasma-derived ctDNA was characterized in 227 plasma samples from 39 patients with metastatic/advanced malignancies (Fig. 1, Table 1). The cohort under evaluation here consisted of 9 major cancer categories: non-colorectal gastrointestinal cancer (*n* = 11), gynecologic cancer (GYN, *n* = 7), genitourinary cancer (*n* = 5), colorectal cancer (CRC, *n* = 4), head and neck cancer (H&N, *n* = 4), skin cancer (melanoma *n* = 3; cutaneous squamous cell carcinoma, *n* = 1), cancer of unknown primary (CUP, *n* = 2), breast cancer (*n* = 1) and lung cancer (*n* = 1) (Table 1**;** Supplementary Fig. 1A). All patients had locally advanced or metastatic disease at treatment initiation, and the study population had a median age of 62 years (range: 38-82), with 56% females. Four out of 39 (10%) patients were identified as microsatellite instability-high (MSI-H). Tumor mutational burden (TMB) ranged from 0.66 to 81.48 mutations/Megabase with a median of 4.27 mutations/Megabase. All patients received immunotherapy ± targeted therapy or chemotherapy. Treatment was evenly distributed between regimens: immunotherapy alone (31% [12/39]), immunotherapy combined with chemotherapy (26% [10/39]), immunotherapy plus targeted therapy (23% [9/39]), and immunotherapy combined with both targeted therapy and chemotherapy (21% [8/39]) (Supplementary Fig. 1A). There were no significant correlations between cancer category and treatment (Fisher’s exact test *P* = 0.12), age at start of the treatment (ANOVA P = 0.60) or sex (Fisher’s exact test *P* = 0.32). The patient’s best overall response (BOR) to therapy varied, with 37% (14 of 39) attaining partial response (PR), 53% (20 of 39) maintaining stable disease (SD; *n* = 8 patients remained SD > 6 months), and 11% (4 of 39) progressive disease (PD).

**Table 1:** Patient characteristics (*N* = 39)

Characteristic		
Age at the start of therapy (years)	Median (range)	62 (38 to 82)
Sex, *n* (%)	Female	22 (56.4)
	Male	17 (43.6)
Stage at initial cancer diagnosis, *n* (%)	III	6 (15.4)
	IV	33 (84.6)
Treatment type, *n* (%)	IO (immuno-oncology)	12 (30.8)
	IO+chemotherapy	10 (25.6)
	IO+targeted	9 (23.1)
	IO+targeted+chemotherapy	8 (20.5)
Cancer categories, *n* (%)	Non-CRC gastrointestinal cancer^a^	11 (28.2)
	Gynecologic cancer	7 (17.9)
	Colorectal cancer	4 (10.3)
	Genitourinary cancer	5 (12.8)
	Head and neck cancer	4 (10.3)
	Skin cancer^b^	4 (10.3)
	Unknown primary cancer	2 (5.1)
	Breast cancer	1 (2.6)
	Lung cancer	1 (2.6)

^a^Non-CRC gastrointestinal cancer included anus, appendix, biliary tract, esophagus, pancreas, stomach.

^b^Skin cancer included melanoma (*n* = 3) and squamous cell carcinoma (*n* = 1).

The median plasma sample count for each patient was 5, with a maximum of 12 samples collected (Fig. 1**;** Supplementary Fig. 1A). ctDNA was positively detected from 2.04-239,315 PPM (tumor fraction ≈ 0.0002%-23.9%), with 33% of detections occurring in the ultrasensitive range below 100 PPM (tumor fraction ≈ 0.01%; Supplementary Fig. 1B). ctDNA levels varied with the tissue of origin of the primary tumor (Supplementary Fig. 1B), with the highest median ctDNA detection observed in GYN cancer (24,313 PPM) and the lowest median ctDNA level detected in breast cancer (2.75 PPM).

### Early on-treatment ctDNA correlated with clinical characteristics

Initial (early on-treatment) plasma samples were collected following the initiation of the treatment of interest (median: 4 days) in 97% (36/37) of patients. ctDNA was detected in 86% of early on-treatment samples (32/37), with 19% (6/32) of the detections below 100PPM, and detection status was not significantly associated with cfDNA input amoung (Supplementary Fig. 2A, B). ctDNA levels at the early on-treatment timepoint varied among different cancer groups with the highest and lowest median ctDNA detected in gynecologic and breast cancer, respectively (Supplementary Fig. 2A). Of note, in 6 out of 9 cancer categories, the ctDNA detection rate at the early on-treatment timepoint was 100% (breast, CRC, GYN, H&N, lung and cancer of unknown primary; Supplementary Fig. 2C); 3 out of 5 early on-treatment ctDNA-negative patients underwent prior treatment, possibly contributing to absence of ctDNA signal. Significantly higher early on-treatment ctDNA level was observed in patients with BOR of PD relative to non-PD patients (Supplementary Fig. 2D).

### Early changes in ctDNA status are highly prognostic of patient outcome

**Fig. 2:**
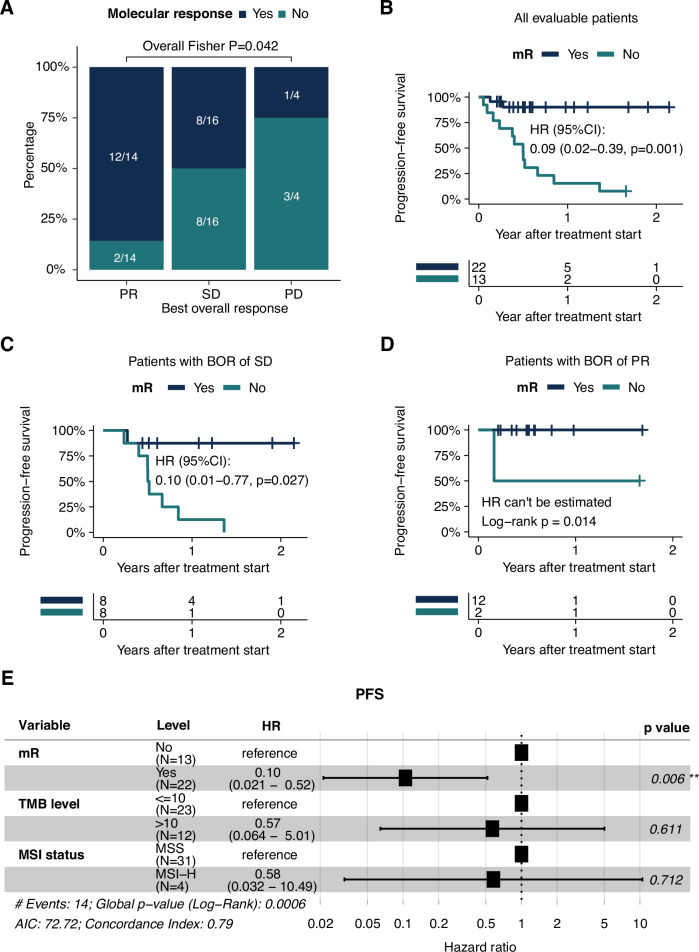
Early ctDNA dynamics are prognostic of patient outcome. **A** Association between early ctDNA dynamics and best overall response (*N* = 34). P values were calculated using Fishers’ exact test. Bars are colored by molecular response (mR) status (blue = mR, teal = no mR). **B** Kaplan–Meier (KM) curve of PFS in patients with mR (blue) and without mR (teal) (*N* = 35). mR was defined as >50% ctDNA reduction in ctDNA levels between the 2nd (median 23 days after the initial plasma sample; IQR: 21 to 29 days) and the initial plasma sample timepoint. HR, corresponding confidence intervals and P values were calculated using Cox regression. **C** KM curve of PFS in the subgroup of patients with a BOR of SD (*N* = 16), stratified by mR (blue, 8/16) or no mR (teal, 8/16). HR, corresponding confidence intervals and P values were calculated using Cox regression. Two patients with BOR of SD were not evaluable due to missing the initial plasma sample samples required to determine mR status. **D** KM curve of PFS in the subgroup of patients with a BOR of PR (*N* = 14), stratified by mR (blue, 12/14) or no mR (teal, 2/14). P value was calculated using a log-rank test. **E** Results of multivariable Cox regression analysis (*N* = 35) including early ctDNA dynamics (mR vs. non-mR), TMB ( ≥ 10 Muts/MB vs. <10 Muts/MB) and MSI status (MSI-H vs. MSS).

We assessed the prognostic value of early ctDNA dynamics and hypothesized that a rapid reduction of ctDNA level was indicative of therapeutic response. In this analysis, we evaluated ctDNA dynamics between the initial and the second plasma timepoint, which included samples collected a median 23 days after the initial plasma sample (IQR: 21 to 29 days). Molecular response (mR) was defined as a > 50% reduction in ctDNA levels from the initial to the second plasma timepoint. Early ctDNA mR significantly correlated with best overall response (BOR, Fig. 2A); 86% of PR patients achieved mR compared to 50% of SD and 25% of PD patients (Fisher’s exact test P = 0.042). Consistently, mR was significantly predictive of longer progression-free survival (PFS; HR 0.09, 95% CI, 0.02 to 0.39, P = 0.001, Fig. 2B). We observed numerically longer overall survival (OS) in mR patients compared with those without mR, though this finding requires further validation in larger cohorts (Supplementary Fig. 3A). The prognostic effect of early ctDNA dynamics was robust across various thresholds of ctDNA reduction. When mR was defined at a more permissive (>30%) or more stringent (>75%) level, the association between mR and longer PFS remained significant (Supplementary Fig. 3B and 3C). Of note, we observed a marginally significant OS separation between patients with >75% ctDNA reduction compared to those without (Supplementary Fig. 3D). Time-dependent ROC analyses further validated that mR highly predictive of 2-year PFS and OS events with AUCs of 0.89 and 0.86, respectively (Supplementary Fig. 3E and 3F).

Next, we assessed whether mR could provide additive clinical value to the BOR (in line with RECIST), and putative biomarkers of IO therapy benefit. Among patients with a BOR of SD, patients with mR (8 of 16, 50%) demonstrated significantly longer PFS compared to those who did not achieve mR, demonstrating the capacity of ctDNA to stratify patients with this ambiguous designation (HR 0.10, 95% CI, 0.01 to 0.77, *P* = 0.027; Fig. 2C). The majority of patients with a BOR of PR achieved mR (12 out of 14, 86%) and none of them experienced disease progression; in the relatively small subset of patients (*n* = 2) with a BOR of PR who did not have mR, PFS was 50% at 1 year of follow up (Fig. 2D). Furthermore, multivariable analyses demonstrated that mR was independently prognostic of PFS when adjusted by TMB level and MSI status (Fig. 2E).

### Longitudinal ctDNA status is associated with clinical outcome

**Fig. 3:**
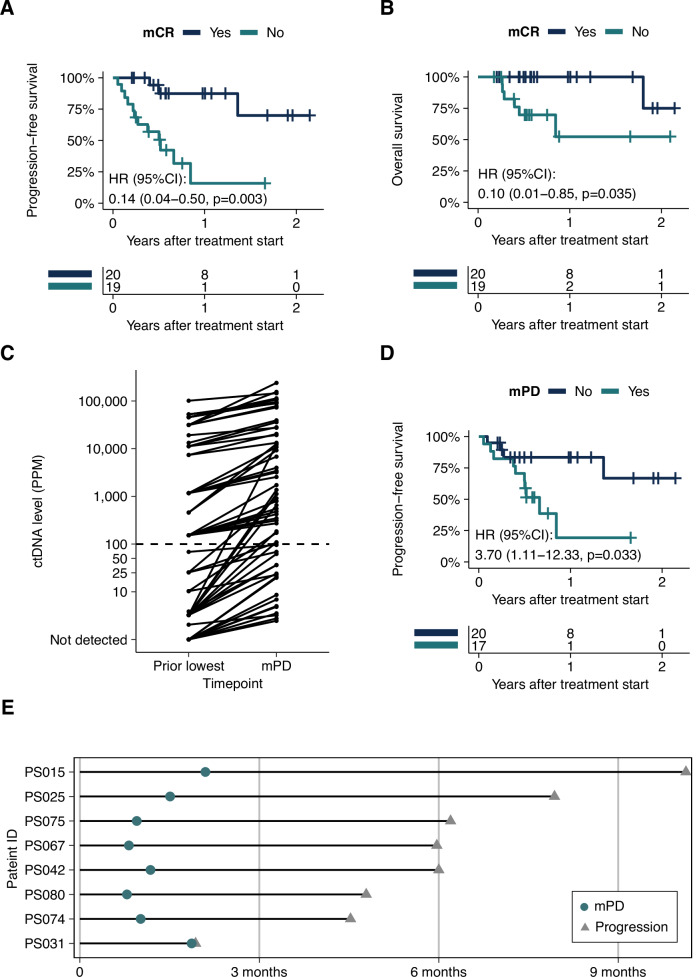
Longitudinal ctDNA status is prognostic of clinical outcome. **A**, **B** Kaplan–Meier (KM) curve of PFS/OS in patients with mCR (blue) or without mCR (teal) (*N* = 39). mCR was defined as any ctDNA clearance during follow up. HR, corresponding confidence intervals and P values were calculated using Cox regression. **C** ctDNA level of mPD plasma samples (*N* = 63) and corresponding previous lowest timepoint. mPD is defined as ctDNA level increases of ≥30% relative to the lowest ctDNA level in previous timepoints. **D** KM curve of PFS in patients with mPD (teal) or without mPD (blue) (*N* = 37). HR, corresponding confidence intervals and P values were calculated using Cox regression. **E** Swimmer plot showing the mPD sample timepoint (teal circle) relative to the clinical progression event (grey triangle) (*N* = 8).

We next leveraged longitudinal ctDNA profiles to stratify patients into groups with or without any ctDNA clearance (molecular complete response, mCR) during followup. Patients that achieved mCR showed markedly higher survival rates of both PFS and OS relative to those who never cleared ctDNA (PFS: HR 0.14, 95% CI, 0.04 to 0.50, P = 0.003; OS: HR 0.10, 95% CI: 0.01 to 0.85, *P* = 0.035; Fig. 3A and 3B). We defined patients with ctDNA level increases of ≥30% relative to the lowest ctDNA level in previous timepoints as molecular progressive disease (mPD) patients. Out of 63 mPD timepoints, 27% (17) of mPD events were identified below 100 PPM (Fig. 3C). We observed that if a patient ever had mPD during followup, their probability of disease progression was more than 3 times higher compared to those without mPD (HR = 3.70, 95%CI: 1.11 to 12.33, *P* = 0.033; Fig. 3D). None of the patients with mPD achieved durable long-term survival, underscoring the need to validate mPD calls in larger cohorts. Of note, for patients with any mPD timepoint, the median lead time of mPD is 160.5 days (range: 2 to 246 days) compared to PD identified by standard of care imaging (median time from treatment start to clinical, imaging-based progression: 182.5 days; Fig. 3E).

**Fig. 4:**
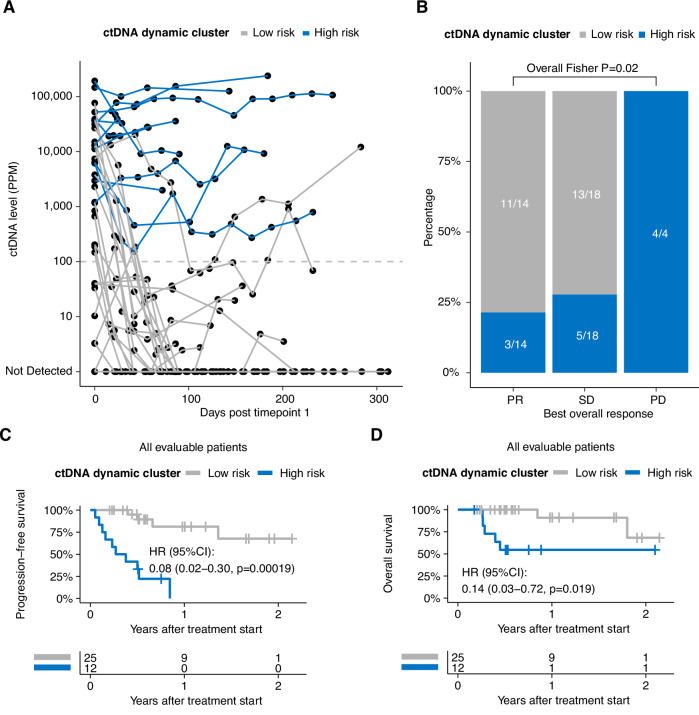
Dynamic clustering of longitudinal ctDNA profiles. **A** Spider plot showing the ctDNA dynamics of patient groups identified by unsupervised clustering. High-risk cluster = blue, low-risk cluster = grey. **B** Stacked barplots depicting the association between ctDNA dynamic clusters and best overall response. P values were calculated using Fishers’ exact test (*N* = 36). High-risk cluster = blue, low-risk cluster = grey. One patient lacked sufficient follow up to assign BOR and was excluded. **C**, **D** Kaplan–Meier (KM) curve of PFS/OS in patient groups identified by unsupervised clustering (low-risk vs. high-risk: grey vs. blue) (*N* = 37). HR, corresponding confidence intervals and P values were calculated using Cox regression.

Next, we applied unsupervised clustering analyses on the longitudinal ctDNA profiles and two groups were identified with distinct patterns of ctDNA dynamics during the course of treatment (Fig. 4A). Generally, patients in cluster 1 (*n* = 12, high-risk) tended to have higher initial ctDNA levels, maintained relatively high ctDNA levels and typically failed to attain ctDNA clearance. Patients in cluster 2 (*n* = 25, low-risk) presented with lower initial ctDNA levels compared to the high-risk cluster, experienced early and rapid decreases in ctDNA levels and maintained undetectable ctDNA status. The identified high-risk ctDNA dynamic cluster had a significantly increased prevalence of patients with a BOR of PD, while patients identified in the low-risk cluster typically achieved a BOR of PR or SD (Fig. 4B). Consistent with early ctDNA dynamics, we observed significantly longer PFS and OS in patients assigned to the low-risk cluster compared with the high-risk cluster (PFS: HR 0.08, 95% CI, 0.02 to 0.30, *P* = 0.00019; OS: HR 0.14, 95% CI, 0.03 to 0.72, *P* = 0.019; Fig. 4C and D). Furthermore, ctDNA dynamic cluster assignment further stratified risk in patients with a BOR of SD (Fig. S4A), suggesting a potentially complementary relationship with imaging-based assessment, although this is exploratory work that requires further validation. Of note, low risk ctDNA dynamic cluster was an independent prognostic factor (HR = 0.11, 95%CI: 0.013 to 0.98, *p* = 0.048) to PFS to mR and mCR in a multivariable Cox regression. To assess the importance of ultrasensitive detection, we performed a sensitivity analysis by setting a detection threshold of 100 PPM for ctDNA measurements. When samples with ctDNA levels below 100 PPM were classified as undetectable, the clustering analysis failed to identify patient subgroups with statistically significant differences in clinical outcomes (Supplementary Fig. 4B and 4C).

## Discussion

Immunotherapy has fundamentally transformed the therapeutic approach to advanced-stage cancer management. Despite this, durable clinical benefit remains limited to 20-40% of patients, and we require new biomarkers beyond conventional measures such as MSI status, PD-L1 and TMB to improve predictive accuracy for patient selection^1–3^. Non-invasive approaches including liquid biopsy have shown promise in addressing this significant clinical challenge, with both early ctDNA dynamics and serial ctDNA monitoring emerging as powerful prognostic markers^12–14^.

Early identification of disease progression is vital in clinical practice as it enables the initiation of alternative treatment or the escalation of current therapies. Our study demonstrates that early decreases in ctDNA within approximately 3 weeks of treatment initiation correlate with significantly improved clinical outcomes in patients receiving immunotherapy. Specifically, patients attaining mR had significantly improved PFS, and presented a 75% lower rate of disease progression (Fig. 1; 1 year progression probability for patients with/without mR: 9.9% vs. 84.6%; HR = 0.09, P = 0.001) compared to patients who did not attain mR at 1 year. Notably, several patients with partial response had shorter survival than those with stable disease. PR patients without molecular response had significantly worse outcomes than those achieving mR, indicating that radiographic shrinkage without molecular clearance may reflect incomplete disease control with residual active disease undetected by CT imaging. Furthermore, mR effectively stratified patients with a BOR of partial response or stable disease into distinct prognostic groups. Discordance between imaging and ctDNA responses showed no unifying clinical pattern, suggesting biological heterogeneity rather than measurement bias. Notably, patients with stable disease who achieved mR demonstrated superior 2-year progression-free survival (87.5% vs 28.7% for the overall cohort), suggesting ctDNA identifies clinical benefit despite absent radiographic shrinkage. However, instances of transient molecular response in radiographically progressive disease emphasize the continued need for serial monitoring. Importantly, mR remained independently prognostic for PFS in a multivariable analysis, after adjusting for TMB and MSI status. These findings support mR as a potential prognostic marker, consistent with standards already adopted for advanced non-small-cell lung cancer^15,16^ and melanoma^17^ patients treated with immune checkpoint inhibitors. Of note, while aligned with standardization frameworks like ctDNA-RECIST, our approach for defining molecular response prioritizes practical applicability by employing fixed percentage thresholds (>50% reduction for mR, ≥30% increase for mPD) rather than non-overlapping 95% confidence intervals. Although CI-based methods offer statistical rigor, our findings indicate that fixed thresholds provide robust prognostic stratification and simpler clinical implementation, warranting future comparative validation.

Longitudinal ctDNA monitoring revealed additional prognostic insights. Patients achieving molecular complete response, which was defined as any ctDNA negative timepoints during follow up, demonstrated longer PFS and OS compared to those with persistent ctDNA signal. Molecular progressive disease (≥30% increase in ctDNA) preceded radiographic progression by a median of **160.5** days, potentially offering an earlier opportunity for treatment modification. Others have suggested that dynamic risk profiling has the potential to inform personalized therapy selection. For example, the dynamic outcome probability model developed by Kurtz et al.^18^, which integrates risk assessments throughout the patient treatment course, demonstrated improved performance compared to pretreatment markers or pathologic response assessment alone. Here, we used unsupervised clustering analysis of ctDNA profiles to identify distinct patterns that correlate with differential survival outcomes. Restricting our model to ctDNA-based inputs alone yielded a more parsimonious model that maintains prognostic utility for long-term outcomes while reducing potential barriers to clinical adoption.

These results align with previous work evaluating the prognostic utility of ctDNA dynamics for response to anti-PD-L1 therapies across various tumor types^9^, though our investigation offers several distinct advantages. We conducted earlier on-treatment assessment (median 23 days), included a broader range of advanced solid tumors and treatment combinations, and employed an ultrasensitive assay that captured ctDNA signal below 100 PPM (**33**% of positive ctDNA detections). Several study limitations merit consideration. While this study utilized plasma for consistency across the pan-cancer cohort, we acknowledge that proximal sampling strategies may offer enhanced sensitivity for specific tumor types. Future investigations that evaluate urine-based ctDNA for genitourinary malignancies or saliva-based monitoring for head and neck cancers could help further improve detection thresholds in localized or low-shedding disease. This study focused primarily on the clinical validity of using ctDNA for monitoring patient response to immunotherapy. Future prospective studies should be performed with strategic sampling at key timepoints to establish clinical utility across treatments and tumor types. The overall sample size was modest (N = 39), with heterogenous tumor types, limiting tumor-specific conclusions. Moreover, patients were treated with various immunotherapy-based approaches including combination therapies. Thus the conclusion drawn here should be further validated in larger independent cohorts, ideally in prospective interventional studies with real-time ctDNA decision-making.

In summary, our findings suggest that ctDNA kinetics could serve as an early response biomarker in the late-stage IO setting, potentially identifying non-responders following one cycle of treatment. This could enable earlier radiological assessment or treatment discontinuation, preventing unnecessary toxicities, and potentially improving patient outcomes. Conversely, the observation of marked ctDNA increases could indicate either escalated treatment or conversion to new therapeutic regimen. While these results support integrating ctDNA kinetics as a biomarker in the advanced IO setting, interventional studies with prespecified cutoffs and real-time analysis are necessary to support clinical implementation.

## Methods

### Study enrollment, patient information and sample analysis

We prospectively enrolled patients with metastatic or locally advanced disease, who had histopathologically confirmed stage III-IV disease at the time of initial diagnosis. Patients were monitored radiologically according to standard of care every 2–3 months, or earlier if clinically indicated. All participants provided written informed consent under protocol approval from the Institutional Review Board of University of California San Diego (No. 130794: UCSD PREDICT [Profile Related Evidence Determining Individualized Cancer Therapy]). All procedures were performed in line with the principles of the Declaration of Helsinki. Patient confidentiality was maintained through de-identification and centralized database monitoring by UCSD. The study incorporated a blinded protocol: Personalis researchers conducted ctDNA analyses without knowledge of patient outcomes or clinicopathological characteristics, while UCSD investigators collected clinical data and samples without access to ctDNA results. All ctDNA analyses were performed retrospectively using prospectively collected samples and clinical follow-up data.

### Plasma isolation

Whole blood specimens were collected in two 10 mL Streck Cell-Free DNA blood collection tubes, and stored at ambient temperature prior to being processed according to manufacturer protocol. Plasma was then stored at -80C until cfDNA extraction was performed. cfDNA was extracted from a median of 3.5mLs of plasma (range: 0.8-4.8mLs), at concentrations ranging from 0.95-14.7 ng cfDNA/mL plasma. Twenty-four hours before cfDNA extraction, plasma samples from identical patient timepoints were thawed, consolidated, and maintained at 4°C. To ensure optimal cfDNA recovery, the consolidated plasma underwent high-speed clarification at 16,000 x g to eliminate cryoprecipitates immediately before extraction using either QIAamp Circulating Nucleic Acid or QIAsymphony Circulating DNA kits (Qiagen).

### Whole genome and circulating tumor DNA sequencing methods

Matched tumor and normal tissue samples underwent WGS to identify somatic variants. Here, tumor specimens were first macrodissected to ensure a minimum tumor cellularity of 20%. Genomic DNA was extracted from these samples using the QIAamp DNA Mini Kit. Library preparation for WGS used the KAPA HyperPrep Kit following shearing of the DNA. Sequencing was performed on an Illumina NovaSeqX platform, targeting 30X depth of coverage.

Somatic variant detection included three main stages: alignment, quality enhancement, and variant detection. Reads were aligned to the hs37d5 reference genome using BWA-mem^19^. The Picard toolkit was used to remove duplicate reads, and the Genome Analysis Toolkit (GATK)^20^ was used for sequence realignment and base quality score recalibration. Single-nucleotide variants (SNVs) were called using MuTect^21^.

For ctDNA analysis, patient-specific hybrid capture probe panels were developed using Personalis’ NeXT Personal platform. The design process involved analyzing the WGS data to target somatic variants in exonic, intronic, and intergenic regions. Variants with allele frequencies over 10% were prioritized. Stringent criteria were used to exclude regions with known germline SNPs, high GC content, or other complexities to enhance detection specificity. The final panels included approximately 1,800 top-ranked somatic variants.

cfDNA was processed using the KAPA HyperPrep Kit before enrichment with patient-specific probe panels. Deep sequencing was then performed on Illumina NovaSeqX instrumentation. The analysis pipeline for ctDNA data included genome alignment and a molecular consensus building protocol to ensure high-fidelity signal detection. ctDNA levels were then quantified in parts per million (PPM) by aggregating tumor-derived signals from the panel targets. Detection status was determined using a one-tailed Poisson test with a significance threshold of p ≤ 0.001 to classify samples as ctDNA-positive or negative.

### Statistical analysis and data management

All analyses were conducted using the R statistical environment (version 4.1.3), with no a priori sample size determination. We employed two-sided statistical tests throughout the analysis unless specifically noted. Unsupervised clustering analysis included only the longitudinal ctDNA levels of each patient. Specifically, each patient was represented with a univariate time series, and the distance between patients was based on the comparisons between time series. Data processing and visualization were executed using a suite of R packages, including tidyverse (v1.3.2) and lubridate (v1.9.2) for data manipulation, while visualization was performed using ggplot2 (v3.4.2), ggpubr (v0.4.0), scales (v1.2.1), and ggnewscale (v0.4.9). Additional methods are provided in Supplementary Notes.

## Supplementary information

Supplementary Information

## Data Availability

Processed patient data is accessible through Zenodo (10.5281/zenodo.17102322).

## Code availability

All code necessary for reproducing the analysis and figures presented in this manuscript are available through the same Zenodo repository (10.5281/zenodo.17102322).
