# Supplementary material for: Ultrasensitive ctDNA monitoring reveals early predictors of immunotherapy response in advanced cancer
Source: NPJ Precis Oncol. 2026 Jan 24;10:79. doi: 10.1038/s41698-026-01287-3 (PMC12936199; doi:10.1038/s41698-026-01287-3)

Supplementary materials

Ultrasensitive ctDNA monitoring reveals early predictors of immunotherapy response in advanced cancer

Daisuke Nishizaki[1,3], Allison Law[1,3], Bailiang Li[2,3], Charles Abbott[2,3], Yi Chen[2,3], Suzanna Lee[1], Rachel Pyke[2], Kathleen Keough[2], Gregory A. Daniels[1], Kay T. Yeung[1], Sean M. Boyle[2,4], Richard Chen[2,4], and Shumei Kato[1,4]

[1]UC San Diego Moores Cancer Center, La Jolla, CA; [2]Personalis Inc., Fremont, CA; [3]These authors contributed equally. [4]These authors jointly supervised.

Corresponding author

Shumei Kato, MD
Associate Professor of Medicine
Division of Hematology and Oncology
Center for Personalized Cancer Therapy
Moores Cancer Center
UC San Diego Health
Email: smkato@health.ucsd.edu

Supplementary notes

Library preparation and whole genome sequencing from matched tumor-normal samples

Formalin-fixed and paraffin-embedded (FFPE) tumor specimens underwent macrodissection to enhance tumor content, with a mandatory minimum tumor cellularity threshold of ≥20% as confirmed by pathological assessment. Tumor specimen were collected from metastatic biopsies in 10 cases (26%) and primary tumors in 29 cases (74%). Paired normal samples consisted of whole blood collected in EDTA tubes. DNA extraction from matched tumor and normal tissue was then performed using optimized protocols with the QIAamp DNA Mini Kit (QIAGEN, Germantown, MD, USA). For WGS library preparation, 100-500 ng of genomic DNA underwent acoustic shearing (Covaris LLC, Woburn, MA, USA), followed by processing with the KAPA HyperPrep Kit (Roche Sequencing Solutions, Pleasanton, CA, USA) using customized methodologies. Libraries were then purified using AMPure XP beads, with subsequent quantification via the KAPA Library Quantification Kit (Roche Sequencing Solutions, Pleasanton, CA, USA). Sequencing was conducted on a NovaSeqX platform (Illumina, San Diego, CA, USA) to achieve 30X depth of coverage.

Somatic Variant Detection Through Paired Tumor-Normal Whole Genome Sequencing Analysis

We implemented a comprehensive genomic analysis pipeline for matched tumor and normal WGS samples, adhering to the Broad Institute's best practice guidelines as previously described[1, 2]. The analysis workflow consisted of three primary stages: alignment, quality enhancement, and variant detection. Initially, BWA-mem[3] aligned individual read pairs to the hs37d5 reference genome. The Picard toolkit[4] (RRID:SCR_006525) was then used to identify and eliminate duplicate reads based on 5' positional comparison of read pairs. Subsequently, the Genome Analysis Toolkit[5] (GATK, RRID:SCR_001876) was used to perform sequence realignment and base quality score recalibration (BQSR). For somatic variant detection, MuTect[6] (RRID:SCR_000559) was used to analyze the paired tumor-normal BAM files to identify single-nucleotide variants (SNVs). We then applied stringent

quality control filtering to the somatic SNV calls, incorporating metrics for local sequence coverage, read quality, strand bias, and statistical assessment of normal sample allele presence.

Design and Implementation of the NeXT Personal Probe Panel

The development of patient-specific hybrid capture probe panels utilized Personalis' NeXT Personal platform, incorporating proprietary algorithms under standardized operating procedures[1, 7]. Panel design began with comprehensive analysis of matched tumor-normal WGS data, targeting somatic variants across exonic, intronic, and intergenic regions. Variant identification employed Mutect (v1.1.6) with default parameters, followed by systematic error rate assignment based on observed substitution patterns in solid tumors. Target selection focused on somatic variants exhibiting allele frequencies exceeding 10%. The platform implemented stringent exclusion criteria to enhance plasma-based detection specificity, removing regions containing known germline SNPs, CHIP variants, high GC content (≥80%), elevated polymorphic rates, mapping complexities, systematic biases, short tandem repeats, and low sequence complexity regions. Variants were then prioritized using a composite ranking system, followed by selection of approximately 1,800 top-ranked genome-wide somatic variants for panel inclusion. Panel manufacturing was then completed by Twist Bioscience (South San Francisco, CA, USA).

Circulating Cell-Free DNA Analysis Using NeXT Personal: Library Preparation, Enrichment, and Sequencing Protocol

Cell-free DNA (cfDNA) processing was conducted in CLIA-certified and CAP-accredited laboratories at Personalis following standardized operating procedures. The protocol utilized cfDNA inputs ranging from 2.17 to 49.55 ng (median: 16.85 ng) for library preparation using the KAPA HyperPrep Kit (Roche Sequencing Solutions, Pleasanton, CA, USA) with optimized methodologies. The variability in library input mass (2.17-49.55 ng; 50 ng maximum allowable input) reflects differences in both the

plasma volume extracted and the cfDNA concentration per mL of plasma across samples. Plasma volumes ranged from 0.8-4.8 mL, and cfDNA yield per mL varied between patients/timepoints (0.95-14.7 ng/mL). We have previously established that cfDNA input quantity does not significantly correlate with ctDNA detection status, ctDNA burden, or assay limit of detection within this input range[7]. Library quantification was performed using a Lunatic spectrophotometer (Unchained Labs, Pleasanton, CA, USA), followed by enrichment using patient-specific NeXT Personal probe panels following theFast Hybridization and Wash Kit (Twist Bioscience, South San Francisco, CA, USA) protocol with proprietary modifications. Post-capture libraries underwent 9 cycles of PCR amplification, with subsequent QC via TapeStation (Agilent Technologies, Santa Clara, CA, USA). Libraries were then purified using AMPure XP beads and quantified using the KAPA Library Quantification Kit (Roche Sequencing Solutions, Pleasanton, CA, USA). Deep sequencing was performed on a NovaSeqX platform (Illumina, San Diego, CA, USA) to maximize unique molecule detection (target coverage: 100,000x).

NeXT Personal cfDNA Analysis Methodology

This study employed Personalis' standardized production pipeline for comprehensive NeXT Personal data analysis[1, 8]. The analytical workflow consists of three primary phases: genome alignment, molecular consensus building, and ctDNA detection. Initial processing begins with BWA-MEM (Burrows-Wheeler Aligner, v1.0.2) alignment of sequencing data to the human reference genome (version hs37d5). The pipeline then implements a sophisticated molecular consensus building protocol to ensure high-fidelity signal detection. Read-pairs are grouped by mapped positions, with additional refinement to prevent false consensus formation. This refinement includes identification of non-reference alleles present across multiple consensus groups, enabling precise molecular differentiation. Stringent quality control measures are integrated throughout the process. These include dual-strand representation requirements, maximum divergence thresholds of 2.5% across

consensus molecules, and a minimum base quality score of 29. Consensus formation requires 90% base-call agreement within molecular groups, with subsequent filtering of reads containing more than 20% masked bases. Consensus reads then undergo secondary BWA-MEM mapping to eliminate alignment artifacts from sequencing errors.

Following noise suppression, ctDNA quantification is performed by aggregating tumor-derived signals across patient-specific panel targets, yielding ctDNA levels in parts per million (PPM) based on unique molecule counts. PPM can be approximately translated to tumor fraction: 1 PPM ≈ 0.0001% tumor fraction. Detection status is determined using a one-tailed Poisson test, comparing observed tumor signals against expected background noise. To maintain analytical specificity exceeding 99.9%, the significance threshold is set at $p \leq 0.001$, definitively classifying samples as ctDNA-positive or negative. By using a p-value-based approach to calling signal detected/not detected, rather than fixed allele frequency thresholds, the method effectively normalizes detection efficiency across diverse genetic alterations, accounting for locus-specific variations in detection sensitivity and blood-based variant frequencies.

Clonal hematopoiesis of indeterminate potential

To address potential interference from Clonal Hematopoiesis of Indeterminate Potential (CHIP), we implemented a comprehensive dual-pronged strategy in our ctDNA assay design. Our primary approach leverages tumor-matched normal variant calling, which effectively distinguishes true somatic variants from CHIP mutations based on their characteristic signal distribution patterns. Since CHIP variants exhibit higher prevalence in normal blood cells compared to tumor tissue, they are systematically filtered out during tumor-normal comparative analysis. Additionally, we exclude commonly documented CHIP-associated genomic regions from our bespoke panel design. This framework ensures robust somatic variant detection while minimizing CHIP-related false positives.

Definition of mR, mCR, and mPD

For each patient, molecular response (mR) was defined as a >50% reduction in ctDNA levels from baseline to the second plasma timepoint. Patient PS047, who was missing baseline plasma but with a ctDNA negative second plasma, was considered as mR. Patient PS070, PS088 and PS033, whose initial and second plasma were ctDNA negative, was considered as mR. Patient PS071 can't be evaluated with mR status and was excluded in the mR analyses due to lack of the second plasma sample. Molecular complete response was defined as any ctDNA negative during the followup. We then defined the molecular progressive disease at the plasma level. For each plasma, if there is a ≥30% increase of ctDNA level relative to the lowest ctDNA level in previous timepoints, such plasma was defined as molecular progressive disease (mPD) timepoint. Patients with any mPD plasma were considered mPD patients. The approaches utilized herein to define mR, mCR and mPD are conceptually consistent with established ctDNA monitoring frameworks[9–12].

Survival and Lead Time Analyses

We conducted comprehensive survival analyses to evaluate overall survival (OS) and progression-free survival (PFS) in all evaluable patients using Kaplan–Meier plots and Cox regression models. OS was measured from treatment initiation until death or loss to follow-up, while PFS was measured from treatment initiation until disease progression. Statistical analyses were conducted using R statistical software, specifically employing Survival (3.3-1), survminer (0.4.9), finalfit (1.0.4), gt (v0.10.1), and mcr (v1.2.2) packages to generate statistical measures including hazard ratios, confidence intervals, and one-year survival probabilities. Inter-group survival differences were evaluated using log-rank tests, while the relationship between continuous variables, particularly ctDNA levels, and survival outcomes was assessed through Cox regression modeling. To establish ctDNA's independent prognostic value, we performed multivariable Cox regression analyses, incorporating key clinical

variables including ctDNA, TMB and MSI status. Lead-time calculations may be susceptible to bias from variability in surveillance imaging intervals and blood collection schedules. To address this, the study design incorporated dense longitudinal sampling and protocol-specified imaging intervals, thereby minimizing potential systematic errors in lead-time estimation.

Supplementary References:

1. Black JR, Bartha G, Abbott CW, et al: Ultrasensitive ctDNA detection for preoperative disease stratification in early-stage lung adenocarcinoma. Nat Med 1–7, 2025

2. Garcia-Murillas I, Abbott CW, Cutts RJ, et al: Whole genome sequencing powered ctDNA sequencing for breast cancer detection. Ann Oncol , 2025

3. Li H: Aligning sequence reads, clone sequences and assembly contigs with BWA-MEM [Internet], 2013[cited 2025 Dec 10] Available from: http://arxiv.org/abs/1303.3997

4. Picard Tools - By Broad Institute [Internet][cited 2025 Dec 11] Available from: https://broadinstitute.github.io/picard/

5. McKenna A, Hanna M, Banks E, et al: The Genome Analysis Toolkit: A MapReduce framework for analyzing next-generation DNA sequencing data [Internet]. Genome Res 20:1297–1303, 2010[cited 2025 Dec 11] Available from: http://genome.cshlp.org/content/20/9/1297

6. Cibulskis K, Lawrence MS, Carter SL, et al: Sensitive detection of somatic point mutations in impure and heterogeneous cancer samples [Internet]. Nat Biotechnol 31:213–219, 2013[cited 2025 Dec 11] Available from: https://pmc.ncbi.nlm.nih.gov/articles/PMC3833702/

7. Northcott J, Bartha G, Harris J, et al: Analytical validation of NeXT Personal®, an ultra-sensitive personalized circulating tumor DNA assay. Oncotarget 15:200, 2024

8. Black JRM, Frankell AM, Veeriah S, et al: LBA55 an ultra-sensitive and specific ctDNA assay provides novel pre-operative disease stratification in early stage lung cancer. Ann Oncol 34:S1294, 2023

9. Jakobsen AKM, Spindler K-LG: ctDNA-Response evaluation criteria in solid tumors – a new measure in medical oncology [Internet]. Eur J Cancer 180:180–183, 2023[cited 2025 Dec 12] Available from: https://www.ejcancer.com/article/S0959-8049(22)01803-2/fulltext

10. Zhang Q, Luo J, Wu S, et al: Prognostic and Predictive Impact of Circulating Tumor DNA in Patients with Advanced Cancers Treated with Immune Checkpoint Blockade. Cancer Discov 10:1842–1853, 2020

11. Bratman SV, Yang SC, Iafolla MA, et al: Personalized circulating tumor DNA analysis as a predictive biomarker in solid tumor patients treated with pembrolizumab. Nat Cancer 1:873–881, 2020

12. Al-Showbaki L, Wilson B, Tamimi F, et al: Changes in circulating tumor DNA and outcomes in solid tumors treated with immune checkpoint inhibitors: a systematic review [Internet]. J Immunother Cancer 11:e005854, 2023[cited 2025 Dec 12] Available from: https://pmc.ncbi.nlm.nih.gov/articles/PMC9933752/

Supplemental Figure 1 | A. Waterfall plot depicting patient response characteristics, plasma sampling count, and clinical characteristics. **B.** The distribution of all ctDNA detection levels from all patients in different cancer groups. Each dot represents the ctDNA level in any ctDNA+ plasma sample. The box denotes the interquartile range of the distribution, with the middle line denoting the median. Whiskers denote the 5th and 95th percentiles.

A

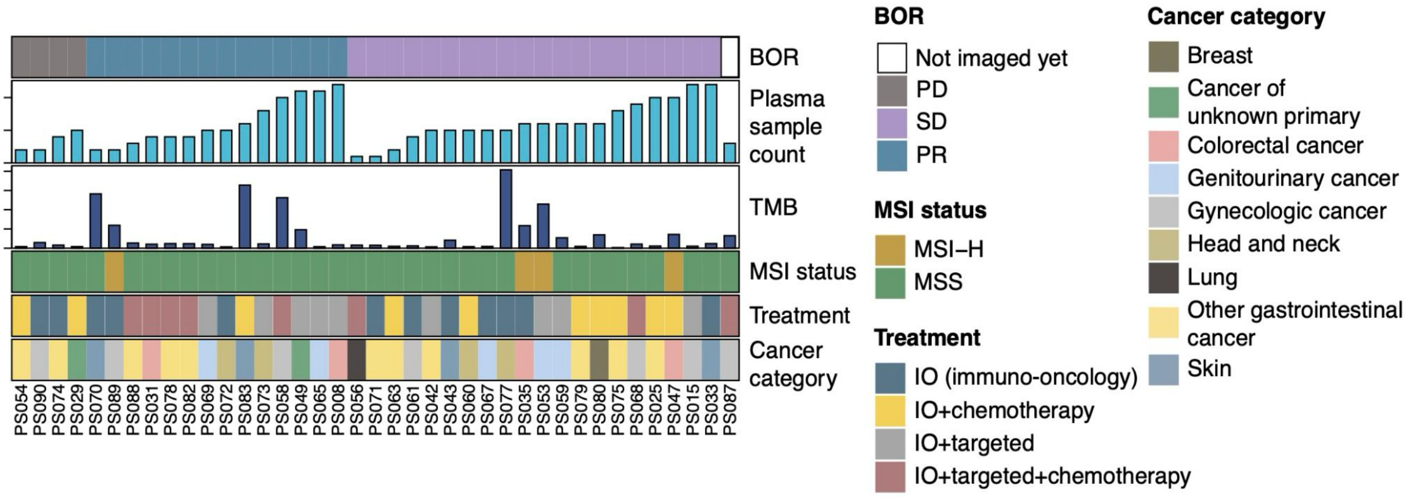

B

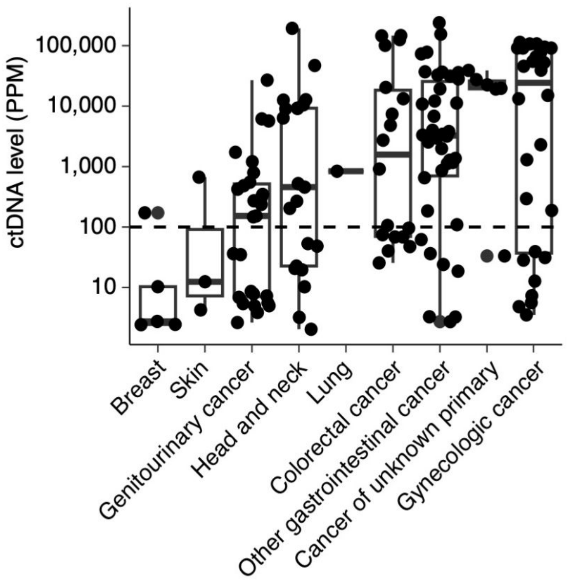

Supplemental Figure 2 | A. The distribution of initial ctDNA levels in different cancer groups. Each dot represents a ctDNA detection in a plasma sample. The box denotes the interquartile range of the distribution, with the middle line denoting the median. Whiskers denote the 5th and 95th percentiles. **B.** cfDNA input amounts into the assay split by initial ctDNA detection status and level. The box denotes the interquartile range of the distribution, with the middle line denoting the median. Whiskers denote the 5th and 95th percentiles. P value was calculated using the Kruskal-Wallis test. **C.** Initial ctDNA detection rates in different cancer groups. Blue represents percent with ctDNA detected, gray represents percent with undetectable ctDNA at the initial ctDNA collection timepoint. **D.** The distribution of initial ctDNA levels in patients with BOR of PD and non-PD. Each dot represents the initial ctDNA level in a patient. The box denotes the interquartile range of the distribution, with the middle line denoting the median. Whiskers denote the 5th and 95th percentiles. P value was calculated using the Student t test.

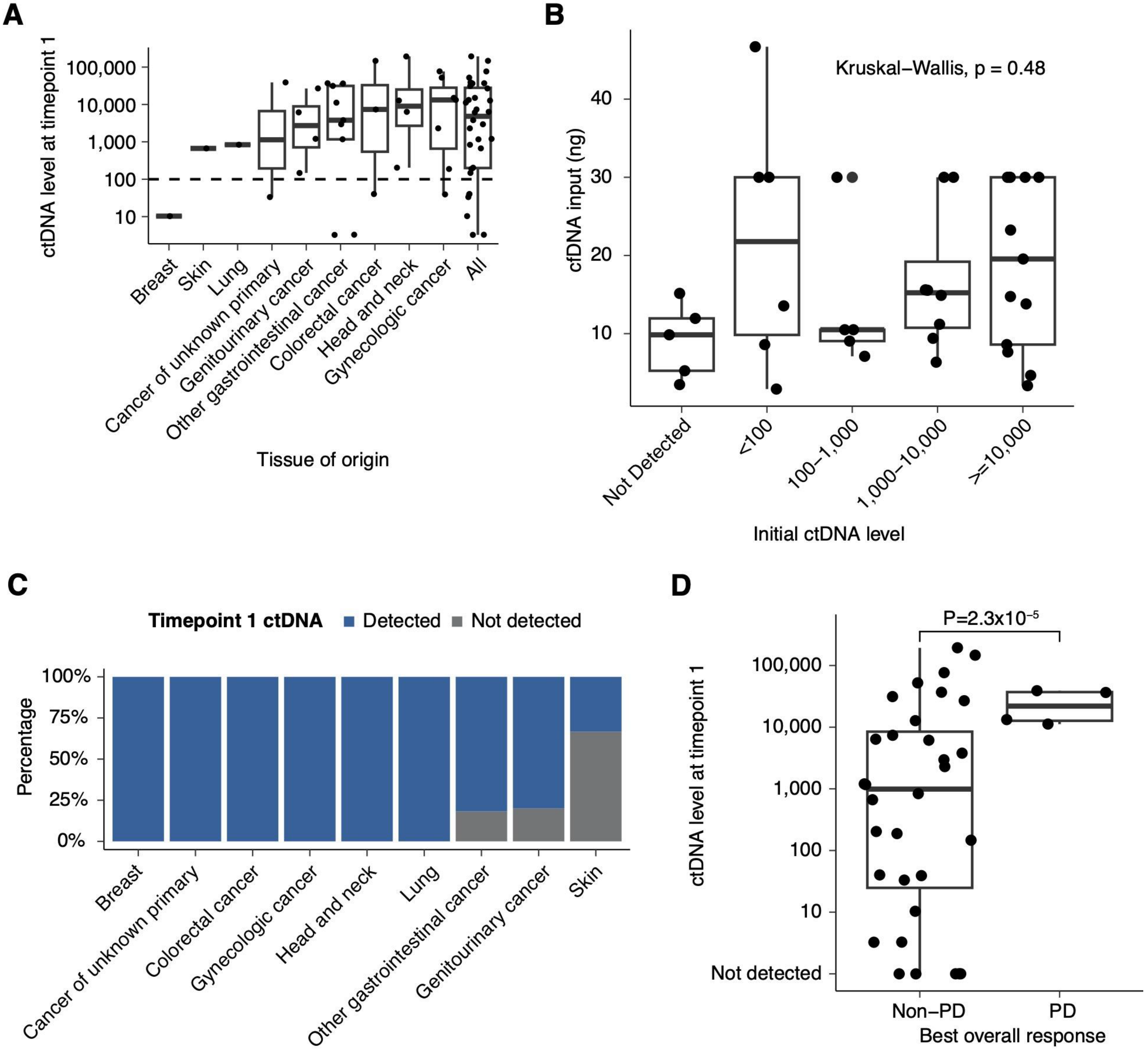

Supplemental Figure 3 | A. Kaplan–Meier (KM) curve of OS in patients with mR (blue) and without mR (teal). mR was defined as >50% ctDNA reduction in ctDNA levels between the 2nd and the baseline timepoint. HR, corresponding confidence intervals and P values were calculated using Cox regression. **B.** KM curve of PFS in patients with mR (blue) and without mR (teal). mR was defined as an alternative value: >30% ctDNA reduction in ctDNA levels between the 2nd and the baseline timepoint. HR, corresponding confidence intervals and P values were calculated using Cox regression. **C,D.** KM curve of PFS (C) and OS (D) in patients with mR (blue) and without mR (teal). mR was alternatively defined as a >75% reduction in ctDNA levels between the 2nd and the baseline timepoint. HR, corresponding confidence intervals and P values were calculated using Cox regression. **E,F**. Time-dependent ROC of ctDNA changes between timepoint 2 and baseline sample with 2-year PFS/OS as end points.

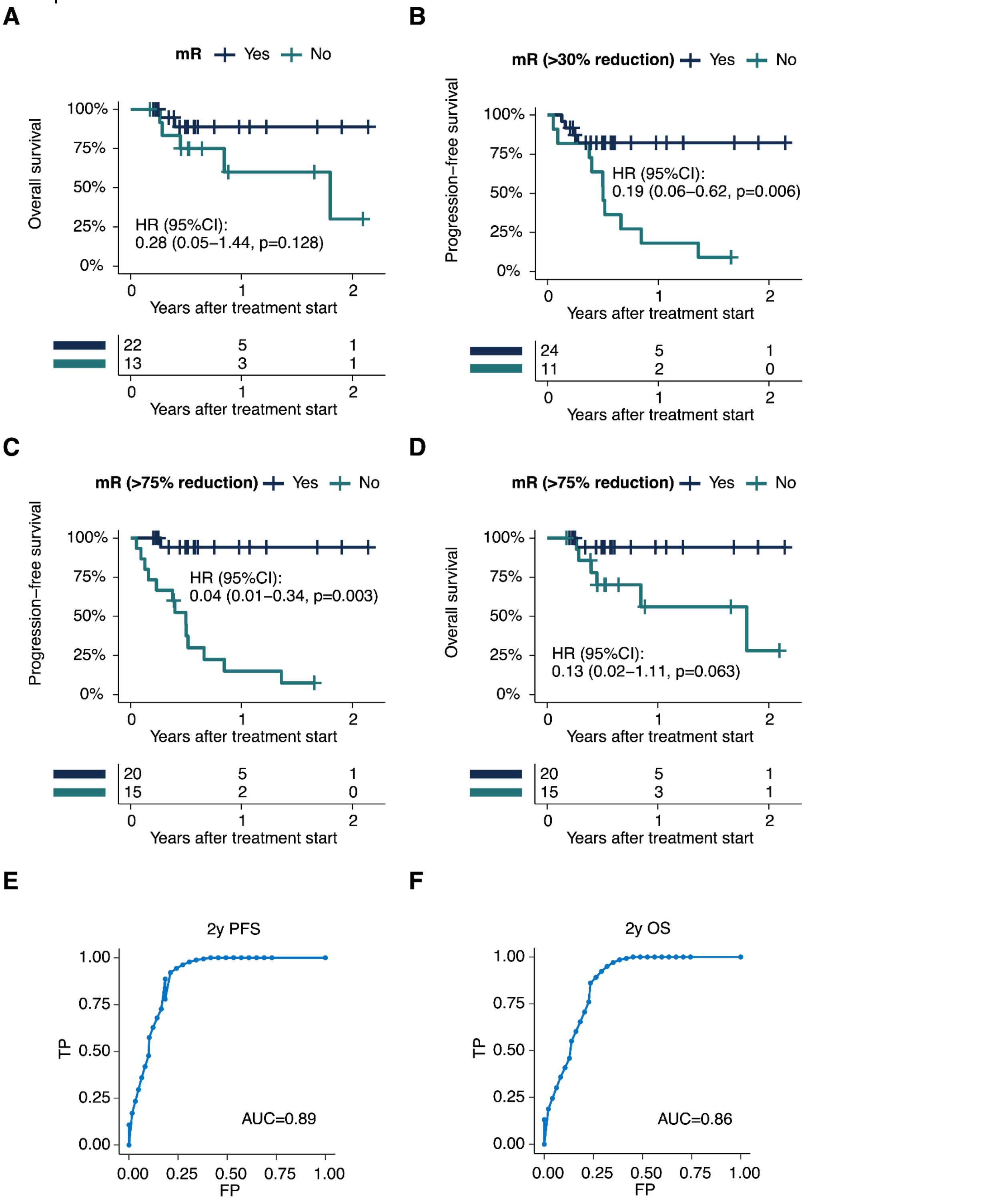

Supplemental Figure 4 | A. Kaplan–Meier (KM) curve of PFS in patients with a best response of stable disease stratified by dynamic ctDNA cluster assignment (low risk vs. high risk: red vs. blue). HR, corresponding confidence intervals and P values were calculated using Cox regression. **B,C.** KM curve of PFS (B) and OS (C) stratified by simulated low-sensitivity dynamic ctDNA cluster assignment, where ctDNA readings below 100PPM are set to undetected (low risk vs. high risk: red vs. blue). HR, corresponding confidence intervals and P values were calculated using Cox regression.

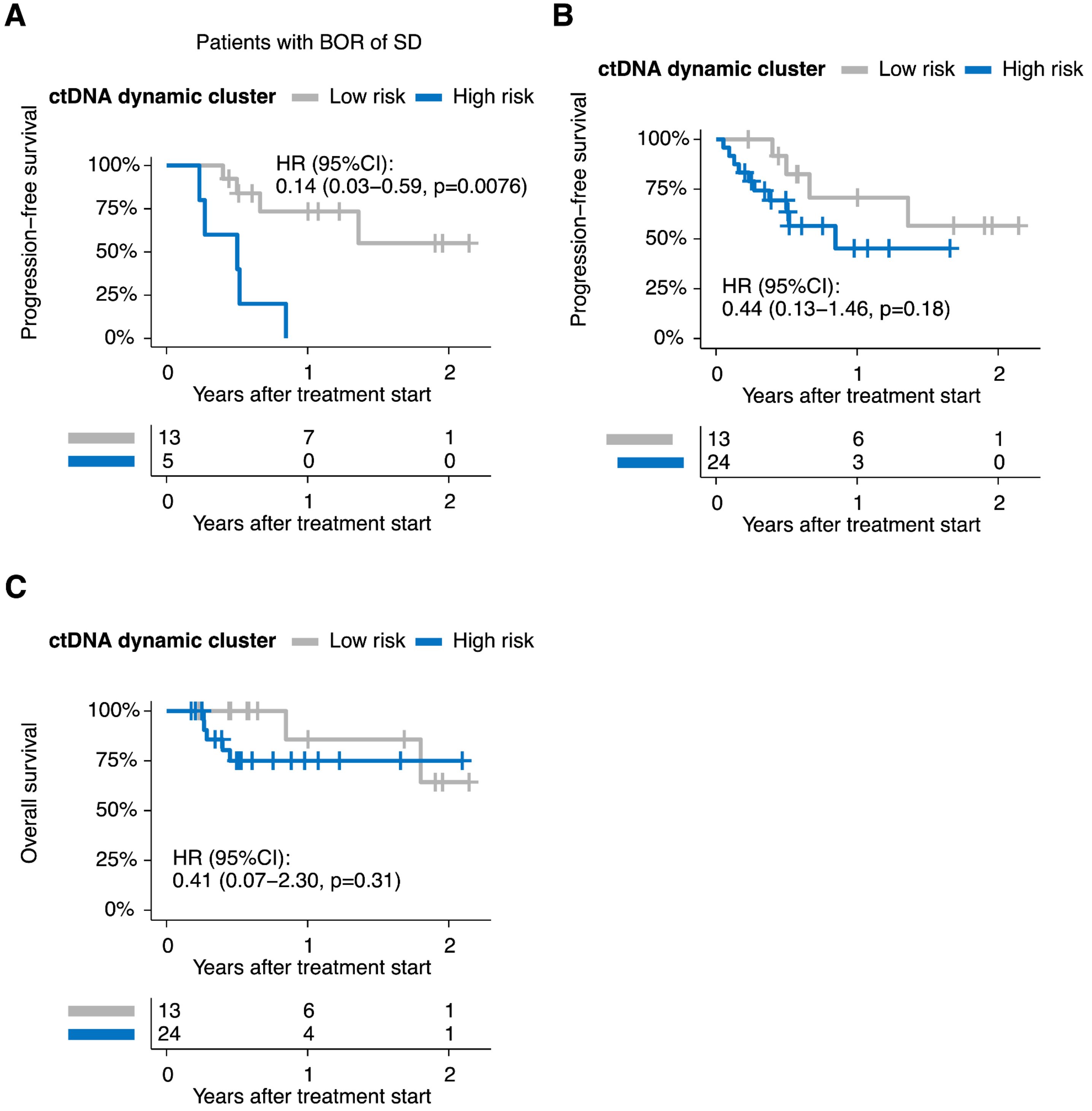

Supplement: Supplementary file 1 — Supplementary Information [file 41698_2026_1287_MOESM1_ESM.pdf]
